# Effect of ethnicity and socioeconomic variation to the gut microbiota composition among pre-adolescent in Malaysia

**DOI:** 10.1038/srep13338

**Published:** 2015-08-20

**Authors:** Chun Wie Chong, Arine Fadzlun Ahmad, Yvonne Ai Lian Lim, Cindy Shuan Ju Teh, Ivan Kok Seng Yap, Soo Ching Lee, Yuee Teng Chin, P’ng Loke, Kek Heng Chua

**Affiliations:** 1 Department of Life Sciences, School of Pharmacy, International Medical University, 57000 Kuala Lumpur, Malaysia; 2 Department of Parasitology, Faculty of Medicine, University of Malaya, 50603 Kuala Lumpur, Malaysia; 3 Department of Medical Microbiology, Faculty of Medicine, University of Malaya, 50603 Kuala Lumpur, Malaysia; 4 Department of Microbiology, New York University School of Medicine, 10010 New York, United States of America; 5 Department of Biomedical Science, Faculty of Medicine, University of Malaya, 50603 Kuala Lumpur, Malaysia

## Abstract

Gut microbiota plays an important role in mammalian host metabolism and physiological functions. The functions are particularly important in young children where rapid mental and physical developments are taking place. Nevertheless, little is known about the gut microbiome and the factors that contribute to microbial variation in the gut of South East Asian children. Here, we compared the gut bacterial richness and composition of pre-adolescence in Northern Malaysia. Our subjects covered three distinct ethnic groups with relatively narrow range of socioeconomic discrepancy. These included the Malays (n = 24), Chinese (n = 17) and the Orang Asli (indigenous) (n = 20). Our results suggested a strong ethnicity and socioeconomic-linked bacterial diversity. Highest bacterial diversity was detected from the economically deprived indigenous children while the lowest diversity was recorded from the relatively wealthy Chinese children. In addition, predicted functional metagenome profiling suggested an over-representation of pathways pertinent to bacterial colonisation and chemotaxis in the former while the latter exhibited enriched gene pathways related to sugar metabolism.

Gut microbiota composition and dynamics are among the main determinants of human health. Gut microbiota plays an important role in nutrient metabolism and innate immune responses^1^,^2^,^3^ and the imbalance of the gut microbiota composition has been shown to be associated with the development of diseases such as inflammatory bowel disease, obesity, and cancer^4^,^5^,^6^. Therefore, understanding how gut microbiota varies across populations will provide a proxy to evaluate the disease susceptibility of different populations and to facilitate the development of population-targeted medicine.

Indeed, evaluation of gut microbiome has been one of the highlights of the human microbiome project (HMP)^7^. However, since the project focused mainly on the western populations, current understanding on gut microbiota distribution was inevitably focusing on the European and American enterotypes^8^. Relatively little is known about the gut microflora composition of the Asian or African populations, especially those in the developing or less developed countries. Interestingly, even when the data is available, the gut flora composition was usually compared across nations with drastic lifestyle and diet difference such as between rural African and European, aborigine Malaysian and New Yorker, cross-European and cross-Asia^9^,^10^,^11^,^12^. Despite providing important baseline information, such comparison is usually masked by the underlying variation in climates, environment, diet and geographical isolation. Further, the age-group studied were usually focused on the adults and infants but not children of pre-adolescent and adolescent years. Such lack of information is perhaps surprising given the strong contribution of gut microbiota to the mental and physical growth during early adolescence^13^,^14^.

In this study, we examined the gut microbiota composition of Malaysian school children (7–12 years old). Our subjects covered three ethnicities namely the Malays, Chinese and Orang Asli (Temiar tribe). To control for the environmental and geographical differences, sampling was carried out in two closely located districts with similar levels of economic development (rural).

## Results

### Socio-economic and hygiene practice of the study group

Significant differences in the demographics and hygiene habits (Pseudo-F = 30.62, *P*_MC_ = 0.001) were detected among the three studied ethnic groups. A follow-up pairwise comparison showed significant difference between all ethnic pairings (*P*_MC_ = 0.001). Generally, the Chinese recorded the highest socio-economic status with no family earning less than RM500 per month (Table 1). Additionally, they exhibited the lowest level of parasitic infection rate (5.9%, single infection; 0% mixed infection) and highest level of hygiene consciousness (i.e. all Chinese subjects practiced proper rubbish disposal and defecate as well as taking bath in the bathroom). A slightly lower household income (~8% <RM500) and higher parasitic load (12.5%) was recorded among the Malays. In contrast, Orang Asli was the poorest group with 45% of the families earning less than RM500 per month. Children under this group were all tested positive for different types of parasitic co-infections such as *Ascaris, Trichuris*, hookworm or/and protozoan parasite infections.

### Terminal Restriction Fragment Length Polymophism (TRFLP)-inferred faecal bacterial richness and composition

**Figure 1:**
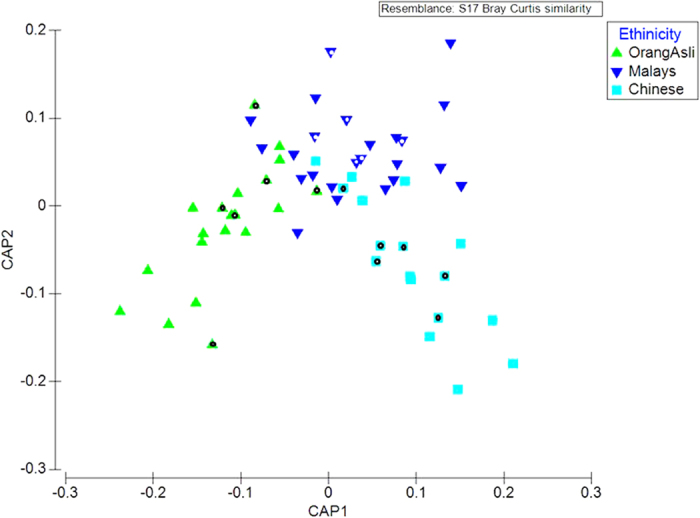
Canonical analysis of principal coordinates (CAP) for TRFLP data. A 67% correct prediction rate was recorded based on the cross validation. Note: Dotted samples were chosen for 16S NGS analysis.

Alpha diversity measures including observed Terminal Restriction Fragments (TRFs) and Shannon Diversity Index suggested that the Chinese harboured a significantly lower faecal bacterial richness in comparison to the Malays and Orang Asli (Table S1). However, no significant differences in TRFs richness and evenness were observed between the two latter groups. We subsequently compared the beta diversity (community overlap) using PERMANOVA and CAP analyses. Both marginal and pairwise PERMANOVA achieved statistical significant at P < 0.01, indicating the presence of ethnicity-specific bacterial composition. Consistent result was observed in the CAP ordination (Fig. 1). Interestingly, Orang Asli cluster was found to be more distantly related to Chinese than Malays in CAP1 axis which coincided with the socio-economic status of the subjects.

### Correlation of social demographic parameters and faecal bacterial diversity

The distribution of the faecal bacterial diversity was significantly modelled using the pre-assigned socio-economic- and hygiene practice-related parameters (Table 2). The former attributed for 15% and the latter 18% of the total variation. When the data was analysed by considering each parameters independently, the step-wise selection algorithm selected “water storage” and “parasitic infection” as the best explanatory parameters for the faecal bacterial community composition. Cumulatively, the two variables explained 12% of the total variation in the model. On the other hand, when we modelled the faecal bacterial composition by splitting the data into each respective ethnic group, “pet” and “water storage” were shown to exert the largest effect on Orang Asli while no parameter was implicated for Chinese and Malay groups.

### Pattern in taxonomic composition inferred using 16S rDNA-based Next Generation Sequencing

**Figure 2:**
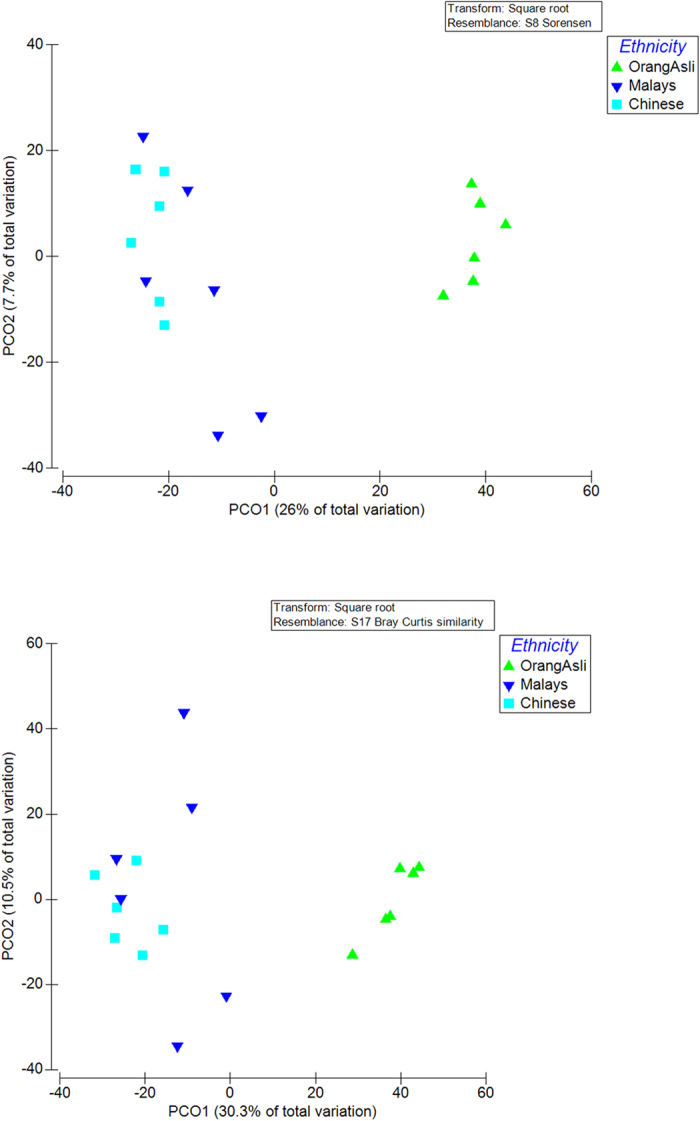
Principle coordinate analysis of 16S NGS sequences distribution (square root transformed) based on (**a**) Sorensen (membership) and (**b**) Bray-Curtis similarity (composition) index.

**Figure 3:**
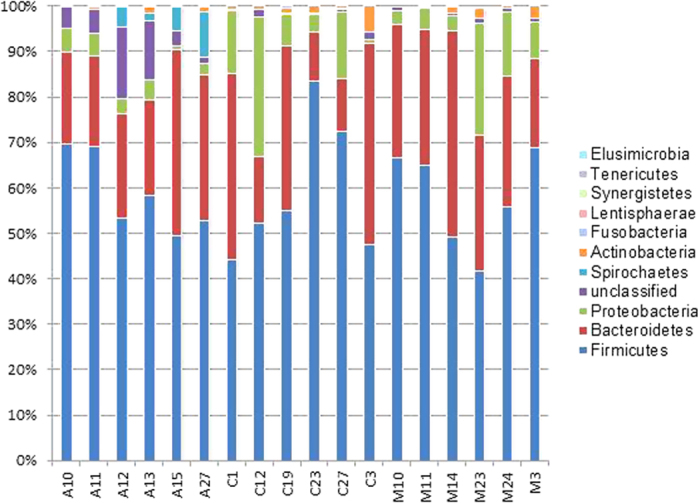
Bacterial phyla distribution inferred based on 16S-based pyrosequencing. The distribution was obtained through sub-sampling of 2503 sequences (the lowest obtained sequences in a sample) across all samples.

In order to examine the taxonomic composition of the faecal sample in detail, 6 replicates from each group were randomly selected for 16S pyrosequencing (see Fig. 1). From a total of 73,934 filtered sequences, 4,787 unique sequences were detected. Sample coverage for all samples was in the range of 97–99% (Fig. S1). Using a 97% sequence homology cut-off, significantly higher OTUs were detected in Orang Asli samples as opposed to Malays and Chinese (Table 1). Additionally, the alpha diversity measures applied here suggested a greater sequence richness and evenness in Orang Asli samples in comparison to others. The beta diversity measures including AMOVA (Table S3) and PCO (Fig. 2) further suggested that the Orang Asli encompassed a distinct community structure and composition. Overall, *Firmicutes* and *Bacteroidetes* were the dominated phyla while *Feacabacterium* and *Prevotella* are the dominant genera in the faeces of the studied cohort (Fig. 3, Fig. S2). However, the differences in faecal microbiota between Orang Asli against Chinese and Malay children can be attributed to the differences in *Aeromonadales*, and unclassified order under *Bacteroidetes* and *Deltaproteobacteria*, and unclassified genus under *Ruminococcaceae* (Table 3). Interestingly, no significant difference in bacterial taxonomic composition was detected between Malays and Chinese despite relative higher socioeconomic status and hygiene awareness in the latter in comparison to the former.

### Predicted functional discrepancy between samples

**Figure 4:**
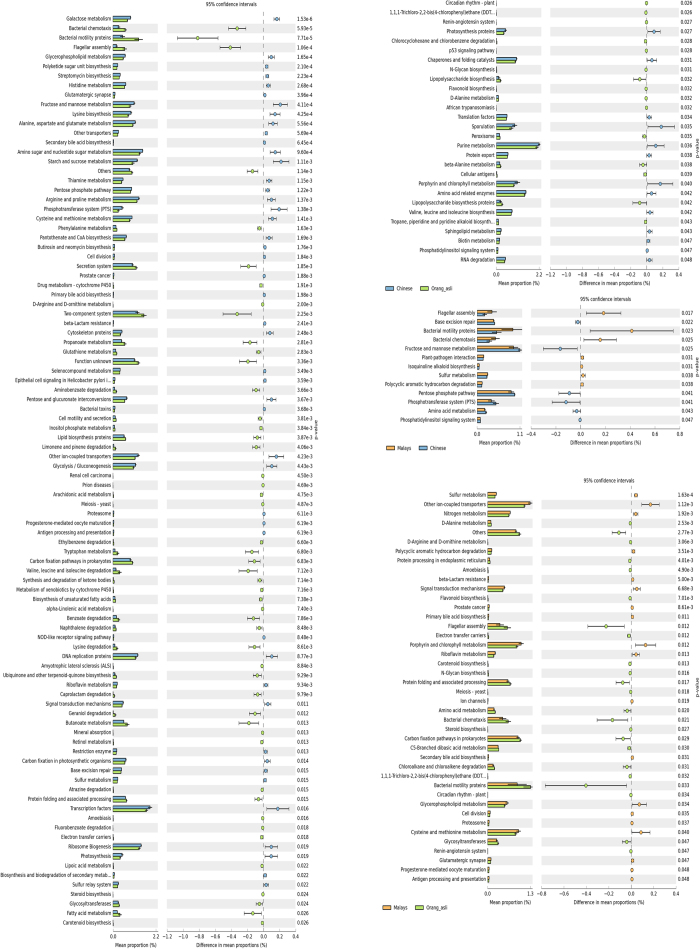
Pairwise comparison of the predicted functional profiles. Note: Only statistical significant features (P < 0.05) were included in the figure.

The faecal bacterial composition was used to estimate the functional difference in the children across three ethnic groups. As expected, gut microbiota in Orang Asli children was predicted to perform more unique functions in comparison to Chinese and Malay children (Fig. S3). From a total of 328 KEGG features, 13 were significantly different between Chinese and Malays, 42 between Malays and Orang Asli, and 124 between Chinese and Orang Asli (Fig. 4). Within the selected KEGG functions, we found 32 features shared by Malays and Chinese which were distinct from the Orang Asli. These included pathways related to bacterial colonisation such as bacterial chemotaxis, flagella assembly, and bacterial motility proteins which were also enriched in the Orang Asli. Conversely, elevated protein and fat related metabolism pathways including bile acid biosynthesis, and amino acid and protein metabolism were detected in both Malays and Chinese. Finally, the functional profiles also suggested that the Chinese might have a higher capability in sugar anabolism and catabolism as several features related to the function was significantly higher in Chinese in comparison to the others.

## Discussion

In this study, we demonstrated that the gut microbial composition of pre-adolescence in Malaysia can be stratified according to the relatively homogenous socioeconomic variation represented by different ethnicity. The pyrosequencing further suggested that the Malays and Chinese gut microbiome shared greater bacterial genetic lineages in comparison to Orang Asli (Fig. 3, Table 3). Interestingly, this pattern coincided with the host genomic characteristic of the studied cohort. Jinam *et al*.^15^ showed that the indigenous Temiar tribe included in this study is genetically distinct to both the Chinese and Malays. Whilst host genetic architecture had been shown to contribute to the development of the individual gut microbiome^16^,^17^, inferring the relationship of host genetic and gut microbial composition is difficult owing to the regionalisation of human population genetics, and the compartmentalisation of socio-demographic characteristics and lifestyles of the population.

In a parallel study focusing on South East Asian children, Mah *et al*.^18^ detected a greater faecal bacterial diversity in children from rural south Thailand while a lower diversity was observed in the urbanised children from Singapore. Similar findings were found to be consistent when the comparison was made between the gut microbiome of urbanised European or American children with those from less developed country such as Bangladesh^11^, Burkina Faso^12^, Malawi^9^ and Venezuela^9^. Our findings are incongruent with these previous findings whereby a higher bacterial diversity was recorded in the Orang Asli in comparison to the Malays and Chinese (Table S1, pyosequencing – all alpha diversity indexes). The Orang Asli was, in general, socially marginalised in Malaysia^19^. Approximately 45% of Orang Asli from this study has a household income of <RM500 per month, much lower than the poverty line income of RM764 (US$254) set by the Malaysian government^20^. A combination of low income and low parental education attainment might have resulted in reduced appreciation to hygiene practices among Orang Asli children in comparison to Malays and Chinese. Based on the DISTLM analysis, both socio-economic- and hygiene-related parameters were found to be significantly related to the gut microbiota diversity (Table 2).

The contribution of gut microbiome dynamic to host immune modulation and responses are well recognised^21^. Various studies had reported strong link between parasitic infection and the changes in gut microbial diversity and community composition. For instance, a significant increase in the *Ruminococcaceae* was observed in population with whipworm (*Trichuris sp*) infection as opposed to the normal population^10^. Separately, elevated representation of *Lactobacillaceae* was found in the ileum of mice infected with *Heligmosomoides polygyrus*^22^. Hayes *et al*.^23^ established that the commensal microflora may play a part in the physiological development of the parasite in the host environment. Based on these, Lee *et al*.^10^ postulated that the elevated gut microbial diversity in Orang Asli as opposed to urbanised population from United States of America might be associated with the high parasitic infection rate in the former. Our results partially corroborated with this findings as the presence of parasitic infection was found to exert a significant (*P* = 0.011) but small influence (explained ~5% variance) onto the gut microbial diversity. Additionally, we also detected an increased in 16S signature of the unclassified genus affiliated with *Ruminococcaceae* but not *Lactobacillaceae* in Orang Asli children.

It is also interesting to note that low gut microbial diversity signature had been detected in individuals with inflammatory bowel disease, metabolic disorder and obesity^5^,^24^,^25^. Nevertheless, differences detected here is unlikely to be related to obesity as the overall prevalence rate for overweight children is similar for both Malays and Chinese (Malays: 9.3–11.6%; Chinese: 6.6–11.6%)^26^,^27^. No data is available for Orang Asli children but the estimated obesity rate for the adult of the same indigenous tribe (Temiar) is comparable (10.1%)^28^.

Overall, bacterial phyla distribution was consistent among the three studied ethnic groups. *Firmicutes* (41–83%) and *Bacteroidetes* (11–45%) made up the bulk of the total detected 16S rRNA gene sequences (Fig. 3). These two phyla had been shown to be the main constituent of gut flora across different populations of different age and nationality^17^,^29^,^30^. The *Firmicutes*/*Bacteroidetes* ratio of the gut microbes in Malays, Chinese and Orang Asli ranged between 2.0 to 3.5 and was comparable to that of the Italian (2.8) and Korean (2.95) but greater than Mongolian (0.77) and African (0.47). High F/B ratio had been previously associated with obesity and high fat diets^6^,^12^,^31^. However, recent publications suggested that such link is over-simplified^32^,^33^,^34^. Owing to the diverse functional capability of each phylum, variation in diets might not induced uniform phylum-wide responses^35^.

A detailed inspection of the taxonomic composition of the gut flora showed that the Orang Asli children harboured significantly greater *Aeromonadales*, unclassified order affiliated to *Bacteroidetes* and *Deltaproteobacteria*, and unclassified genus under *Ruminococcaceae* when compared to the Malays and Chinese (Table 3). These lineages included anaerobic relatives such as *Ruminobacter amylophilus*^36^*, Prevotella ruminicola*^37^*, Ruminococcus flavefaciens*^37^,^38^ etc that are commonly involved in degradation of dietary fibre into short chain fatty acid (SCFA) in human gut. Indeed, vegetables and grains formed a larger proportion of the Orang Asli diet than that of the Malays and Chinese^39^,^40^. Additionally, higher prevalence of *Bacteroidetes* (e.g. *Prevotella*) and *Ruminococus spp*. were also apparent when comparing the gut microbiome composition of populations under the Eastern/African versus Western lifestyle^9^,^10^,^11^,^12^. Interestingly, although the Malays showed a higher frequency in seafood intake than Chinese (70% of the former had fish the day before faecal collection as opposed to 0% in the latter), no significant difference in gut bacterial lineage was detected. Despite the spatial proximity and relatively similar level of district development (rural) of all three ethnicity, the Malays and Chinese children had a higher exposure to westernised and processed diet, coupled with a greater tendency of sedentary lifestyle. In support to previous studies that lifestyle affects gut microbiota diversity and distribution^12^,^18^,^41^; we further showed that the effect is detectable in populations under similar environments with low level of lifestyle variation. It is noteworthy that 16S rRNA gene signature related to *Spirochaetes* was only exclusively found in the Orang Asli children. De Filippo *et al*.^12^ suggested that *Treponema spp*. under *Spirochaetes* might be part of the gut commensals in rural Africans, who consumed high dietary fibre, in order to degrade undigested complex carbohydrates. Nevertheless, as the taxon was also the causative agent of Yaws disease which was common in Malaysian indigenous population previously^42^, the findings warrant a detail evaluation on the possible re-emergence of the disease in Malaysia.

Based on the predicted metagenome profiles (Fig. 4), it was apparent that the Chinese children harboured significantly more genes related to sugar metabolism as opposed to others. The metabolism of sugar is an important pathway for energy harvesting and essential to physical and mental growth in children. In addition, the gut floras of Chinese and Malays also showed greater capability in fat digestion (i.e. primary and secondary bile acid biosynthesis). The elevated pathways related to sugar and fat metabolism corresponded to higher calories intake of the two ethnic groups in comparison to Orang Asli^40^. It is perhaps surprising however to note that the Chinese children, despite enriched energy metabolism pathways, are generally less obese in the later development stage (adulthood) than the other two groups^43^. In comparison, pathways related to bacterial proliferation and colonisation were significantly higher in the Orang Asli. Since no obvious disease symptom was observed from the Orang Asli children (e.g. diarrhoea), the signatures might be sourced from non-pathogenic infection of environmental bacteria, given the lower hygiene consciousness of the indigenous population. These environmentally-acquired bacteria might then form part of the natural innate immune responses on gut surfaces^44^.

In conclusion, using a combination of TRFLP profiling and pyrosequencing techniques, we have showed a close relation between the ethnicity related socioeconomic disparity and gut microbial composition in Malaysian children. Such observation might be related to the host genetic and lifestyle variation. We further highlighted the taxonomic and functional distribution differences across the three ethnic groups. As the first targeted report on Malaysian children gut microbiome, our study contributed to the understanding of the least assessed Asian gut microbiota composition. In addition, the results can be used as a baseline to decipher the link between the dynamics of gut microbial and ethnic-related health risk in Malaysia^43^,^45^.

## Methods

### Sample collection and ethical clearance

A total of 61 faecal samples were collected from primary school children aged 7–12 (10.57 ± 0.35) from rural areas of Perak, Malaysia. None of the subjects were siblings or undergoing antibiotic treatment. The children were given the stool containers a day earlier. They were advised to defecate directly into the tube or pass stool into large clean container and then transfer the specimen into collection tube immediately using the scoop attached to the screw capped container (with an adult’s help). Any faecal sample that contacted with urine was excluded. The faecal samples were transferred to University of Malaya (UM, Kuala Lumpur) within 24 h. All collected samples were preserved in potassium dichromate and stored at 4 °C during transportation.

Specifically, the Malays (n = 24) and Chinese (n = 17) subjects were from Pangkor Island, Manjung while the Orang Asli (indigenous Temiar tribe, n = 20) originated from Bota, Perak Tengah. This village is located approximately 60 km from the town area of Sungai Siput, in the northern part of the interior highlands of upper Perak state (ie., Pos Piah). Pangkor Island and Bota were approximately 50 km away from each other. The main economic activities of Malays and Chinese were fishing and fishing related businesses while the Orang Asli generally practices a self-sustenance lifestyle. Approval of the ethics application with appropriate experimental protocols was obtained from the Medical Ethics Committee (MEC), University of Malaya. All the methods used in this study were in accordance with the approved guidelines (MEC references 824.11 and 920.16). Prior to sample collection, a short briefing pertinent to the study objectives and sampling procedures was given to the volunteers. Sample was only collected from subjects who provided written consent given by their parents/guardians. To record the socio-demographic and sanitation characteristics, each volunteer was requested to fill in a survey form with the assistance of the respective school teachers. Upon arrival in UM, each sample was divided into two tubes: one for microscopic examination of parasites which was stored at 4 °C while the other tube was stored at −20 °C for further DNA extraction.

### Parasitic screening and identification

For parasite identification, formalin-ether concentration technique was employed. Briefly, approximately 1 g to 2 g of stool sample was mixed with 7 ml of 10% formalin and 3 ml ethyl acetate prior to centrifugation at 980 rfc for 5 mins. Wet faecal smear was made from the sediment, stained with 0.85% iodine and observed under the microscope magnification of 100X and 400X for intestinal parasites (i.e., helminths and protozoa). In addition, detection of *Cryptosporidium* oocyst was done by staining faecal smears with Ziehl-Neelsen and examined microscopically (400x magnification).

### DNA extraction and terminal restriction fragment length polymorphism (TRFLP) procedures

DNA extraction was carried out using NucleoSpin Soil kit (MACHEREY-NAGEL, Germany) according to the manufacturer protocol. PCR amplification for TRFLP was conducted using universal bacterial primers 27F and 1492R under thermoprofile described by Chong *et al*.^46^. Both primers were tagged with the fluorescence dye phosphoramidite fluorochrome 5-carboxyfluorescein (FAM) and 6-carboxy-hexachlorofluorescein (HEX) at 5′ end respectively. The amplicons were digested using a 4-base cutter *M*spI (Promega, USA) before subjected to electrophoretic separation using ABI 3100 and ABI 3730XL genetic analysers (Applied Biosystems, USA). ROX labeled GeneScan 500 control was used as the size standard. The resulting fragment profiles were scored, aligned and noise filtered using the web-based programme T-REX^47^. Noise filtered procedure was carried out by removing peaks which are lower than the overall standard deviation. In addition, alignment was carried out by binning the peaks into TRFs with the clustering threshold of 0.8 bp, starting from the smallest peak among all the profiles^48^.

The TRFLP data obtained from T-REX was exported into PERMANOVA + add-on of the PRIMER6 multivariate data analysis package (Plymouth Marine Laboratory, UK) for analyses. Alpha-diversity indices including Shannon diversity index (H’), Inverse Simpson diversity index (1/λ) and Pielou’s measure of species evenness (J’) were calculated using the DIVERSE option. Beta diversity of the gut microbiota was analysed using permutational multivariate analysis of variance (PERMANOVA) and canonical analysis of principal coordinates (CAP)^49^. Both analyses were performed based on the Bray-Curtis Similarity matrix. The PERMANOVA was carried out to test for gut microbial compositional difference between the groupings while the CAP was used to project statistical axes which maximized the difference between the three ethnicity groups. The significance of the PERMANOVA and CAP were calculated based on 999 permutations. To account for the small sample size for multiple group comparison, significance value for PERMANOVA was corrected using Monte Carlo correction. The power of the PERMANOVA was estimated based on approach reported by Hoffmann *et al*.^50^. In brief, the mean composition for each TRFs was first calculated using the actual data and simulated 500 times using Dirichlet-Multinomial distribution assuming both high and low spread (i.e. theta = 0.05 and 0.005 respectively). The number of times simulated P value falls below alpha (0.05) was recorded. According to the simulation (Table S3), the power was between 85–100%.

### 16S rRNA gene-based pyrosequencing

From the 61 extracted DNA samples, 6 replicates of DNA were selected randomly from each ethnic group for 16S-based pyrosequencing. The average age of the selected sample according to ethnicity was 11.0, 10.0 and 10.8 respectively for Malays, Chinese and Orang Asli children. In addition, all Orang Asli children were infected with parasites, while only one individual was infected (subject M24) among the Chinese and Malays. Variable 16S rDNA region V3-V5 was amplified based on barcoded bacterial universal primers 357F (CCTACGGGAGGCAGCAG) and 926R (CCGTCAATTCMTTTRAGT) recommended in the 16S pyrosequencing protocol by the Human Microbiome Project Consortium^51^. The pyrosequencing produced a total of 141,648 raw reads with an average read length of 483 bp. The raw sequences were processed using Mothur software^52^ according to the standard operating procedure described by Schloss *et al*.^53^. Briefly, the raw sequences was denoised using PyroNoise algorithm^54^ and filtered by selecting sequences with minimum length of 200bp and maximum homopolymer of 8 bp. The resulting sequences were aligned in reference to SILVA taxonomy reference file release 119 and sequences with <2 bp difference were merged. Subsequently, chimeric and ambiguous sequences affiliated to plastid, chloroplast, mitochondria and unknown lineages were removed using “chimera.uchime” and “remove.lineage” commands respectively. Finally, the dataset was clustered into operational taxonomic units (OTUs) using a 97% sequence homology cut-off. The final aligned dataset contained 73,934 sequences with a mean length of 404 bp. The raw sequences were submitted to NCBI database under Bioproject PRJNA269514.

Alpha diversity indices for the NGS dataset was calculated by importing Mothur OTUs shared file into PRIMER programme package (Plymouth, UK). To increase the weightage of the rare OTUs, the same shared file was squared-root transformed and used to generate Jaccard and Bray-Curtis similarity matrices. The faecal bacterial assemblage pattern in respect to the two similarity matrices was then ordinated using principle coordinate analyses (PCO). Significant difference in the faecal bacterial composition was detected using analysis of molecular variance (AMOVA). Separately, taxonomic unit showing significantly different representation across the ethnic groups was resolved using METASTATS^55^. Both AMOVA and METASTATS were available in the Mothur programme package.

### Predicted functional profiling using PICRUSt

The relative abundance of gene pathways (classified under Kyoto Encyclopedia of Genes and Genomes (KEGG) database) in each faecal microbiome was predicted using PICRUSt analysis. The prediction was enabled by inferring the gene content and functions reported by the closest reference genome of the bacterial lineages detected in the sample^56^. Prior to the estimation, the taxonomy of the 16S rDNA dataset was classified in reference to the GreenGene database May 2013 release. The associated biome file was generated using “make.biome” command under Mothur programme package, pre-treated using online galaxy terminal and imported into statistical analysis of taxonomic and functional profiles (STAMP) software^57^. Overall, relationship of the functional profiles was illustrated with principle component analysis (PCA) plot. In addition, two-sided Welch’s T-test was conducted to elicit the pathways showing distinct expression patterns across different ethnic groups.

## Additional Information

**Accession numbers**: The sequences reported in this paper were 393 deposited in the NCBI database under Bioproject PRJNA269514.

**How to cite this article**: Chong, C. W. *et al*. Effect of ethnicity and socioeconomic variation to the gut microbiota composition among pre-adolescent in Malaysia. *Sci. Rep*. **5**, 13338; doi: 10.1038/srep13338 (2015).

## Supplementary Material

Supplementary Information

## Figures and Tables

**Table 1:** Socio-economic characteristics and hygiene level of rural children aged 7–12 in Pangkor and Bota, Perak, Malaysia.

	Malays		Chinese		Orang Asli		Total
	Sum	*%*	Sum	*%*	Sum	*%*	
Socioeconomic characteristics							
Ethnicity	24	*39.3*	17	*27.9*	20	*32.8*	61
Gender							
*Male*	15	*62.5*	8	*47.1*	10	*50*	33
*Female*	9	*37.5*	9	*52.9*	10	*50*	28
Income							
*<RM500*	2	8.3	0	*0*	9	*45*	11
Family Size *≥* *5 members*	18	75	1	*5.9*	10	*50*	29
Rear pet(s)	9	14.8	0	*0*	19	*95*	28
Hygiene level							
Parasitic Infection							
*Singl*^a^	2	8.3	1	*5.9*	0	*0*	3
*Mixed*^b^	1	4.2	0	*0*	20	*100*	21
Pipe water availability^c^	24	100	17	*100*	16	*80*	57
Water storage							
*Plastic bottles*	17	70.8	0	*0*	10	*50*	27
*Bucket*	6	25	1	*5.9*	10	*50*	17
*Cement water tank*	1	42	16	*94.1*	0	*0*	17
Cover water storage	20	83.3	1	*5.9*	20	*100*	41
Bathing locations							
*Toilet*	23	95.8	17	*100*	0	*0*	40
*River*	1	4.2	0	*0*	20	*100*	21
Defecation locations							
*Toilet*	24	100	17	*100*	5	*25*	46
*Bushes/river*	0	0	0	*0*	15	*75*	15
Proper rubbish disposal	23	95.8	17	*100*	15	*75*	55

^a^*Trichuris tichiuria* only.

^b^Combination of at least two of the following: *Trichuris trichiura*, *Iodamoeba butschlii*, *Blastocystis* spp., *Entamoeba histolytica*, *Entamoeba coli*, *Ascaris lumbricoides*, and hookworm.

^c^Pipe water supply to the household.

**Table 2:** Distance-based linear models of socioeconomic and hygiene characteristics of faecal bacterial composition in children.

Marginal Tests (Grouped variables)							
Group	SS(trace)	Pseudo-F	P	Prop.			
Socioeconomic	16346	1.6534	0.014	0.155			
Hygiene	19159	1.6836	0.012	0.181			
Marginal Tests (Individual variables)*							
Variable	SS(trace)	Pseudo-F	P	Prop.	Group		
Water storage	8651.9	5.2806	0.001	0.082	H		
Cover water storage	7830	4.7387	0.001	0.074	H		
Parasitic infection	4793.4	2.8133	0.014	0.046	H		
Rear pet	4855.4	2.8515	0.014	0.046	S		
Bathing locations	4261.5	2.488	0.018	0.040	H		
Ethnicity	4178.6	2.4376	0.024	0.040	S		
Family size	4416.9	2.5827	0.024	0.042	S		
Defecation locations	3353.1	1.9402	0.044	0.032	H		
Sequential Tests							
Overall							
Variable	AIC	SS(trace)	Pseudo-F	P	Prop.	Cumul.	res.df
Water Storage	453.66	8651.9	5.28	0.001	0.08	0.08	59
Parastic infection	453.25	4071.8	2.55	0.01	0.04	0.12	58
Orang Asli							
Variable	AIC	SS(trace)	Pseudo-F	P	Prop.	Cumul.	res.df
Pet	148.64	4359.8	2.84	0.049	0.14	0.14	18
Water Storage	148.43	2893.7	1.99	0.067	0.09	0.23	17
Malays/Chinese							
Variable	None						

^*^Only elements with significant effect are included.

**Table 3:** Taxonomic identity of gut microbial taxa showing significant difference in abundance across Malay, Chinese and Orang Asli children.

	Differential abundance taxonomic unit Phylum/Order/Genus				
Malays VS Chinese	None				
Orang Asli VS Malays	Phylum	Mean in Orang Asli	Mean in Malays	p-value	q-value
	unclassified bacteria	0.072	0.008	0.005	0.002
	*Spirochaetes*	0.036	0	0.017	0.004
	Order	Mean in Orang Asli	Mean in Malays	p-value	q-value
	unclassified *Bacteroidetes*	0.116	0.004	<0.001	0.009
	unclassified *deltaproteobacteria*	0.001	0	0.001	0.010
	*Bacteroidales*	0.143	0.301	0.002	0.014
	*Aeromonadales*	0.005	0	0.005	0.021
	unclassified bacteria	0.067	0.007	0.011	0.041
	unclassified *Clostridia*	0.001	0	0.014	0.041
	*Selenomonadales*	0.006	0.014	0.019	0.050
	Genus	Mean in Orang Asli	Mean in Malays	p-value	q-value
	unclassified *Ruminococcaceae*	0.200	0.037	0	0
Orang Asli VS Chinese	Phylum				
	None				
	Order	Mean in Orang Asli	Mean in Chinese	p-value	q-value
	unclassified *Bacteroidetes*	0.116	<0.001	<0.001	0.004
	unclassified *deltaproteobacteria*	0.001	0	0.001	0.021
	*Aeromonadales*	0.005	0	0.002	0.052
	Genus	Mean in OrangAsli	Mean in Chinese	p-value	q-value
	unclassified *Ruminococcaceae*	0.200	0.029	0	0
	unclassified *Clostridiales*	0.083	0.002	<0.001	0.010
	unclassified *Bacteroidetes*	0.119	0	0.001	0.041
	*Alistipes*	0	0.049	0.001	0.042

Note: only taxonomic units with p-value and q-value ≤ 0.05 were reported.
