# Supplementary material for: Effect of ethnicity and socioeconomic variation to the gut microbiota composition among pre-adolescent in Malaysia
Source: Sci Rep. 2015 Aug 20;5:13338. doi: 10.1038/srep13338 (PMC4542465; doi:10.1038/srep13338)
Supplement: Supplementary Information [file srep13338-s1.doc]

**Effect of ethnicity and socioeconomic variation to the gut microbiota composition among pre-adolescent in Malaysia**

Chun-Wie Chong^1^ , Arine F Ahmad^2^ , Yvonne A.L. Lim^2^ , Cindy S.J. Teh^3^ , Ivan K. S. Yap^1^ , Soo-Ching Lee^2^ , Yuee-Teng Chin^2^ , P'ng Loke^4^ , *Kek-Heng Chua^5^

^1^Department of Life Sciences, School of Pharmacy, International Medical University, 57000 Kuala Lumpur, Malaysia

^2^Department of Parasitology, Faculty of Medicine, University of Malaya, 50603 Kuala Lumpur, Malaysia

^3^Department of Microbiology, Faculty of Medicine, University of Malaya, 50603 Kuala Lumpur, Malaysia

^4^Department of Microbiology, New York University School of Medicine, 10010 New York, United States of America

^5^Department of Biomedical Science, Faculty of Medicine, University of Malaya, 50603 Kuala Lumpur, Malaysia

Correspondence to: Kek-Heng Chua (khchua@um.edu.my)

Table S1. Alpha-diversity measures of children faecal samples derived based on TRFLP and 454 NGS analyses (mean ± SE). Note: different alphabet denoted significant difference at P < 0.05.

|  | Ethnicity | | | ANOVA | |
| --- | --- | --- | --- | --- | --- |
|  | Malays | Chinese | Orang Asli | F | P |
| *TRFLP* |  |  |  |  |  |
| Number of TRF | 312.13^a^ | 167.47^b^ | 243.05^ab^ | 4.44 | 0.02 |
| (S) | ±26.96 | ±39.8 | ±37.51 |  |  |
|  |  |  |  |  |  |
| Species Evennes | 0.830 | 0.801 | 0.846 | 2.34 | 1.06 |
| (J') | ±0.05 | ±0.08 | ±0.06 |  |  |
|  |  |  |  |  |  |
| Shannon Diversity | 4.67^a^ | 3.66^b^ | 4.32^ab^ | 6.04 | <0.01 |
| Index (H') | ±0.11 | ±0.27 | ±0.24 |  |  |
|  |  |  |  |  |  |
| Inverse Simpson | 76.58 | 47.01 | 79.55 | 1.58 | 0.21 |
| Index (1/λ) | ±11.77 | ±15.55 | ±13.96 |  |  |
|  |  |  |  |  |  |
| *16S-based NGS* |  |  |  |  |  |
| Number of OTUs | 123.83^a^ | 104.00^a^ | 188.50^b^ | 25.96 | <0.01 |
| (S) | ±7.08 | ±7.41 | ±10.98 |  |  |
|  |  |  |  |  |  |
| Species Evennes | 0.600^a^ | 0.588^a^ | 0.721^b^ | 5.55 | 0.02 |
| (J') | ±0.04 | ±0.03 | ±0.04 |  |  |
|  |  |  |  |  |  |
| Shannon Diversity | 2.97^a^ | 2.80^a^ | 3.85^b^ | 12.57 | <0.01 |
| Index (H') | ±0.19 | ±0.16 | ±0.11 |  |  |
|  |  |  |  |  |  |
| Inverse Simpson | 8.90^a^ | 8.90^a^ | 22.26^b^ | 9.68 | <0.01 |
| Index (1/λ) | ±2.11 | ±2.64 | ±2.63 |  |  |

Table S2. PERMANOVA on Bray-Curtis similarities for TRFLP derived assemblages of bacterial diversity in faecal of children from different ethnic groups. Information given includes degrees of freedom (df), sum of squares (SS), mean square (MS) and P value under Monte-Carlo correction (P_MC_).

*a. Marginal Test*

| Source | Df | SS | MS | Pseudo-F | P_MC_ |
| --- | --- | --- | --- | --- | --- |
| Ethnicity | 2 | 33013 | 16506 | 8.9205 | 0.001 |
| Residual | 73 | 135080 | 1850.4 |  |  |
| Total | 75 | 168090 |  |  |  |

*b. Pairwise Test*

|  |  | Unique |  |
| --- | --- | --- | --- |
| Groups | t | perms | P_MC_ |
| OrangAsli, Malays | 1.75 | 998 | 0.004 |
| OrangAsli, Chinese | 2.76 | 999 | 0.001 |
| Malays, Chinese | 3.69 | 999 | 0.001 |

|  | **Braycurtis** | | **jclass** |  | **hellinger** |  | **gower** |  | **thetayc** |  | **UnW-UniFrac** |  | **W-UniFrac** |  |
| --- | --- | --- | --- | --- | --- | --- | --- | --- | --- | --- | --- | --- | --- | --- |
|  | **Fs** | **P** | **Fs** | **P** | **Fs** | **P** | **Fs** | **P** | **Fs** | **P** | **Fs** | **P** | **Fs** | **P** |
| A-C-M | 3.48 | <0.001* | 2.21 | <0.001* | 3.21 | <0.001* | 1.56 | <0.001* | 2.54 | <0.001* | 2.54 | <0.001* | 4.20 | <0.001* |
|  |  |  |  |  |  |  |  |  |  |  |  |  |  |  |
| A-C | 5.63 | 0.001* | 3.14 | 0.002* | 5.21 | 0.001* | 1.66 | 0.002* | 4.04 | 0.004* | 3.83 | 0.001* | 7.10 | 0.001* |
|  |  |  |  |  |  |  |  |  |  |  |  |  |  |  |
| A-M | 3.78 | 0.003* | 2.54 | <0.001* | 3.35 | 0.002* | 1.73 | <0.001* | 2.72 | 0.006* | 2.87 | 0.004* | 4.83 | 0.003* |
|  |  |  |  |  |  |  |  |  |  |  |  |  |  |  |
| C-M | 1.13 | 0.32 | 1.03 | 0.31 | 1.22 | 0.19 | 1.02 | 0.35 | 0.81 | 0.50 | 1.07 | 0.22 | 1.19 | 0.32 |

Table S3. Analysis of Molecular Variance (AMOVA).

Table S4. Statistical power of the PERMANOVA simulated using Dirichlet-Multinomial distribution (alpha = 5%). Note: Low spread and high spread was estimated at theta = 0.005 and 0.05 respectively.

|  | Orang Asli VS Malays | | Orang Asli VS Chinese | | Chinese VS Malays | |
| --- | --- | --- | --- | --- | --- | --- |
| Sample Size | Power | Power | Power | Power | Power | Power |
|  | Low Spread | High Spread | Low Spread | High Spread | Low Spread | High Spread |
| 10 | 100 | 67.6 | 100 | 0.998 | 100 | 100 |
| 15 | 100 | 86.4 | 100 | 100 | 100 | 100 |
| 20 | 100 | 96.8 | 100 | 100 | 100 | 100 |
| 30 | 100 | 100 | 100 | 100 | 100 | 100 |
| 50 | 100 | 100 | 100 | 100 | 100 | 100 |

Fig S1. Rarefaction curve of 18 samples selected for NGS.

Fig S2. Top 20 bacterial genera detected from the faecal sample


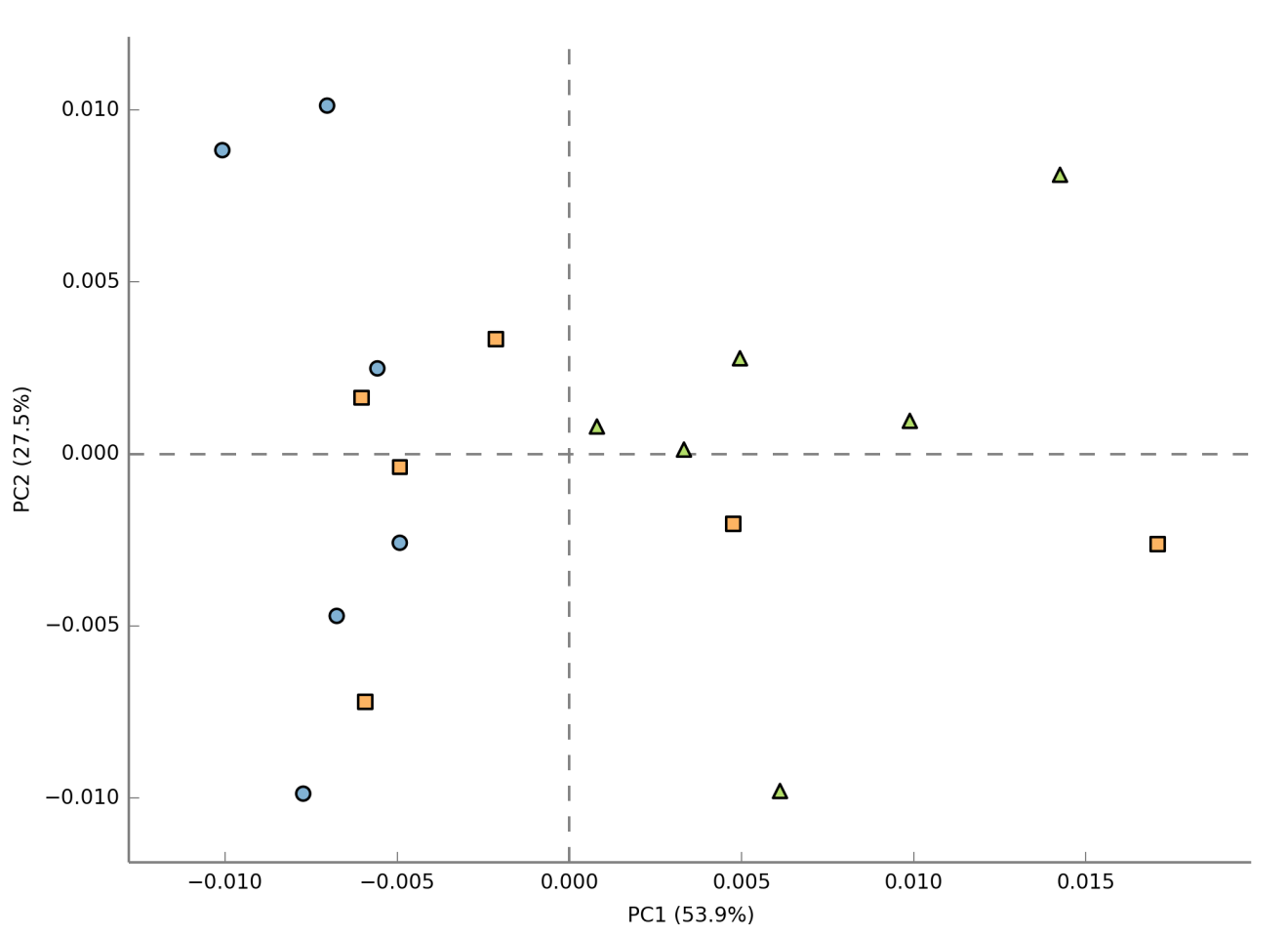


Fig S3. PCA for Malays (turquoise), Chinese (amber) and Orang Asli (green) based on functional differences according to the predicted KEGG Orthologs.
